# Vaccine-Based Immunotherapy for Oropharyngeal and Nasopharyngeal Cancers

**DOI:** 10.3390/jcm14041170

**Published:** 2025-02-11

**Authors:** Daria Maria Filippini, Elisabetta Broseghini, Carlotta Liberale, Giulia Gallerani, Giambattista Siepe, Elisabetta Nobili, Manuela Ferracin, Gabriele Molteni

**Affiliations:** 1 Medical Oncology Unit, IRCCS Azienda Ospedaliero-Universitaria di Bologna, 40138 Bologna, Italy; elisabetta.nobili@aosp.bo.it; 2 Department of Medical and Surgical Sciences (DIMEC), University of Bologna, 40126 Bologna, Italy; g.gallerani@unibo.it (G.G.); manuela.ferracin@unibo.it (M.F.); 3 IRCCS Azienda Ospedaliero-Universitaria di Bologna, 40138 Bologna, Italy; elisabett.broseghin2@unibo.it; 4 Unit of Otorhinolaryngology, Head & Neck Department, University of Verona, 37134 Verona, Italy; carlotta.liberale@gmail.com; 5 Radiation Oncology, IRCCS Azienda Ospedaliero-Universitaria di Bologna, 40138 Bologna, Italy; giambattista.siepe@aosp.bo.it; 6 Department of Otolaryngology-Head and Neck Surgery, IRCCS Azienda Ospedaliero-Universitaria di Bologna, 40138 Bologna, Italy

**Keywords:** virus-related head and neck cancers, oropharynx, nasopharynx, vaccines, immunotherapy

## Abstract

Viral infections such as human papillomavirus (HPV) and Epstein–Barr virus (EBV) play a critical role in the onset of oropharyngeal (OPC) and nasopharyngeal cancer (NPC), respectively. Despite advancements in targeted therapies and immunotherapies, in the recurrent/metastatic setting, these tumors remain incurable diseases with poor prognosis. The development of therapeutic tumor vaccines, utilizing either neoantigens or oncoviral antigens, represents a promising addition to the cancer immunotherapy arsenal. Research on vaccine-based immunotherapy for OPC and NPC focuses on targeting viral antigens, particularly HPV E6/E7 and EBV EBNA1/LMP2. The potential for vaccine platforms, including peptide-based, DNA, RNA, and viral vector-based vaccines, to induce durable immune responses against viral antigens is reported. The early-phase clinical trials evaluating vaccine-based therapies for HPV-related OPC and EBV-related NPC revealed safety and preliminary signs of efficacy; however, further clinical trials are crucial for validation. This review provides an overview of the current landscape of vaccine-based strategies for HPV-related OPC and EBV-related NPC, discussing their biological mechanisms and immune processes involved in anti-HPV and anti-EBV vaccine treatments, with a particular focus on the immune factors that influence these therapies.

## 1. Introduction

### 1.1. Standard of Care for Oropharyngeal and Nasopharyngeal Cancers

Head and neck cancers (HNC) encompass a variety of tumors that occur in different anatomical areas, each exhibiting unique features. The main risk factors for HNC include tobacco smoke, alcohol consumption, and viral infections [1]. Notably, human papillomavirus (HPV) infection is a risk factor for oropharyngeal cancer (OPC), while Epstein–Barr virus (EBV) infection is associated with nasopharyngeal cancer (NPC) [2,3].

Oropharyngeal squamous cell carcinoma (OPSCC) includes cancers of the tonsils, base of the tongue, soft palate, and uvula [4]. Recent statistics indicate that 33% of OPSCCs are HPV-positive [5]. The eighth edition of the American Joint Committee on Cancer (AJCC) staging system distinguishes p16-positive, as a surrogate marker of the presence of HPV, from p16-negative OPSCC to reflect the better prognosis associated with the former [4]. A combination of immunohistochemical (IHC) staining for p16 and in situ hybridization (ISH) for HPV-DNA shows high sensitivity (97%) and specificity (94%) to detect HPV positivity [4].

The treatment modalities for patients with HPV-positive OPSCC generally include surgical excision, radiotherapy (RT), or chemoradiotherapy (CRT) [6], according to disease staging, patient preferences, clinical status, and center expertise. Surgery, particularly for early-stage OPSCC, can involve transoral robotic surgery (TORS), transoral laser microsurgery (TLMS), or traditional surgical methods, depending on the tumor’s size and location [7]. RT is another cornerstone, used either as an exclusive therapy or in the adjuvant setting, alone or with chemotherapy. Exclusive CRT is a common choice based on the patient’s preference or when surgery is not feasible due to factors like large tumor size (T3 or above), poor transoral access, or locally advanced HPV-positive OPSCC [4].

Immune checkpoint inhibitors (ICIs) have been recently evaluated in the locally advanced setting with unsuccessful results [8,9,10]: they have been approved in the first-line setting for recurrent and/or metastatic (R/M) head and neck squamous cell cancer (HNSCC) as well as platinum-resistant R/M HNSCC [11,12].

NPC risk factors include EBV infection, environmental factors (such as diet and smoking), and genetic predisposition. Histologically, NPC is categorized into keratinizing squamous cell carcinoma, consisting of well-differentiated cells producing keratin, differentiated or undifferentiated non-keratinizing carcinoma, and basaloid carcinoma subtypes. Keratinising cancer is more frequent in non-endemic areas, whereas non-keratinising cancer comprises the majority of cases where EBV infection is endemic [13].

Regarding the treatment of EBV-induced NPC, approximately 90% of patients with early-stage disease can be cured by RT alone [14]. RT is the mainstay treatment modality for non-metastatic disease [15]. Nowadays, there is considerable interest in employing proton RT for the treatment of NPC due to its dosimetric advantages [16]. The integration of chemotherapy with RT represents a pivotal advancement in treating locally advanced diseases. Induction chemotherapy serves to enhance distant control and, consequently, overall survival (OS). Patients identified with a high risk of distant metastasis may derive additional benefits from induction chemotherapy in addition to concurrent CRT [13,17].

However, due to the hidden anatomical location of the nasopharynx and atypical early symptoms, most patients with NPC are at stage III or IV at the time of initial diagnosis. Approximately 20–30% of patients with locally advanced NPC fail treatment, primarily due to recurrence and/or metastasis. Immunotherapy has emerged as a promising treatment modality for R/M NPC [17].

However, responses to immunotherapy may vary, with different ICIs and combination strategies playing a critical role in determining its effectiveness [18].

The increasing prevalence of HPV-positive HNC has underscored the need for novel therapeutic approaches, especially given the limitations of traditional treatments such as surgery, RT, and chemotherapy, which can often result in significant morbidity. While HPV vaccination has been well established for cervical cancer prevention, vaccine-based immunotherapies, which have demonstrated promising efficacy in the treatment of HPV-related cervical cancer, are being explored as a potential treatment option for HPV-positive OPC [19]. These vaccines aim to harness the body’s immune system to target and eliminate HPV-infected cells, potentially offering a less invasive and more targeted therapeutic strategy. Given the rising incidence of HPV-related HNC and the proven efficacy of HPV vaccines in cervical cancer prevention [20], there is an urgent need to explore their application in therapeutic contexts for these tumors.

This review highlights key research developments in vaccine-based treatments for OPC and NPC providing perspectives into potential future directions.

We also provide insights into the biological mechanisms of therapeutic cancer vaccines, describing the immune processes induced by anti-HPV and anti-EBV treatments.

### 1.2. Therapeutic Cancer Vaccines

Cancer progression is constantly controlled by the immune system, which can recognize and destroy cancer cells or inhibit metastatic spreading through cell-mediated cytotoxicity. However, cancers can induce a series of alterations in the immune system, leading to an immunosuppressive tumor microenvironment (TME), and resulting in tumor escape and proliferation in cancer cells. 

Tumor cells produce new proteins, mutate, and evade the immune defense mechanisms, leading to “immunoediting”. Cancer immunoediting describes the immune system’s dual role in both protecting the host and shaping the tumor. It is proposed to occur in three phases: Elimination, Equilibrium, and Escape. This process begins after tumor cells undergo a transformation and intrinsic tumor-suppressive mechanisms fail. In the Elimination phase, the immune system actively eliminates the nascent tumor through coordinated actions of innate and adaptive immune responses. If the immune system fails to fully eradicate the tumor, the cancer enters the Equilibrium phase, where tumor growth is suppressed but not eliminated, often for extended periods. The Escape phase, which is often underappreciated, occurs when the tumor adapts to escape immune control. In this phase, tumor cells may recruit immune cells to create an immunosuppressive TME and promote the expression of immune checkpoint molecules. This phase marks the emergence of clinically detectable cancer [21]. A better knowledge of the TME and immune suppressive mechanisms has provided new cancer therapeutic strategies, such as immunotherapy and therapeutic cancer vaccines [22].

A therapeutic cancer vaccine aims to prompt the identification of malignant cells by exposing effective cancer antigens, activating host T cells, and interrupting immune tolerance [23].

### 1.3. Target Antigens

During the process of carcinogenesis, tumor cells acquire genetic alterations that can result in the expression of tumor neoantigens, which are protein/peptide sequences that are expressed specifically by tumor cells. In addition, cancer cells can activate transcriptional programs leading to the expression of embryonic antigens, or unusual amounts of self-antigens, which can both trigger immune recognition. These antigens can be taken up by antigen-presenting cells (APC), including dendritic cells (DCs) and macrophages, and presented to CD4+ T cells leading to their activation and differentiation into effector T cells and memory T cells. If the tumor antigen is presented by MHC I on cancer cells, CD8+ T cells carry out tumor antigen-specific cytolytic activity to eradicate tumor cells [24,25,26].

Since tumor antigens recognized by T cells are central to the efficacy of cancer vaccines, the choice of antigen is extremely important to the efficacy of the cancer vaccine [27]. The ideal antigen for cancer vaccine should be highly immunogenic, necessary for cell survival so that the cancer cannot escape an immune attack by downregulating the antigen, and preferentially expressed in all cancer cells but not normal cells [28].

Both tumor cells and viruses can produce tumor antigens and, according to the cancer antigen source, there are three major origin-based categories of tumor antigens. The first two groups derive from endogenous tumor cells, namely tumor-associated antigens (TAAs) and tumor-specific antigens (TSAs, neoantigens), while the third group derives from exogenous viruses, namely oncogenic virus-derived antigens [29].

TAAs are self-antigens abnormally expressed by tumors and could be expressed in normal tissues. Specifically, TAAs can be classified as overexpressed antigens, differentiation antigens, and cancer testis antigens [30,31]. Overexpressed antigens are derived from proteins that are overexpressed in tumors, but their expression is at much lower levels in normal tissues, while differentiation antigens are expressed only in tumor cells and in the normal tissue of origin. Cancer testis antigens are expressed in tumor cells and cells from adult reproductive tissues, including placenta and testicular cells [30]. The problem with TAAs is the central tolerance, which can hinder the antitumor response of high-affinity T cells against TAAs [32]. The vaccination against TAAs can lead to the onset of autoimmunity reactions.

A TSA is a neoantigen that is expressed only in tumor cells but not normal cells, is typically patient-specific, and arises from genetic mutations. The advantage of TSAs is that they are recognized by high-affinity T cells as non-self antigens since they are not expressed by normal cells. Therefore, TAAs are less likely to be the object of central tolerance and induce autoimmunity, given the specificity of expression [23,33,34].

Based on their expression frequency, neoantigens can be divided into personalized and shared/public neoantigens [23,35]. Personalized neoantigens are identified using high-throughput sequencing of DNA or RNA samples from paired tumors and normal tissues. A fraction of personalized neoantigens comprises public neoantigens that are shared in a subset of patients with a given cancer type. Shared neoantigens are defined as immunogenic epitopes derived from mutations in oncogenes or other hotspot mutations across the genome [29].

Oncoviral antigens derive from viral infections associated with human cancers, including OPC, NPC, cervical carcinoma, hepatocarcinoma, and adult T-cell leukemia [30]. Both neoantigens and oncogenic viral antigens are more immunogenic than TAAs because of the lack of central tolerance and serve as excellent immune system targets to identify and attack cancerous cells.

Classification and characteristics of tumor antigens are described in Table 1.

### 1.4. Vaccine Delivery Platforms

**Figure 1:**
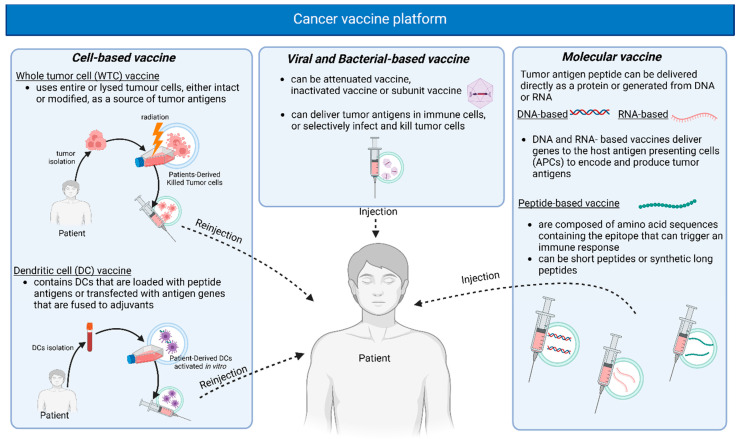
Cancer vaccine platforms are typically categorized into cell-based, viral or bacterial-based, and molecular vaccines.

Developing a therapeutic tumor vaccine using neoantigens or oncoviral antigens is a promising cancer immunotherapy strategy. A significant challenge in effectively developing cancer vaccines is the antigen-delivering system. Cancer vaccine platforms are often divided into cellular, viral bacterial vector, or molecular vaccines, which include DNA, RNA, or peptide-based vaccines [31,36] (Figure 1).

Cellular vaccines can be divided into whole tumor cell (WTC) vaccines, which are based on autologous tumor cells obtained from the patient or cells that are derived from allogeneic tumor cells (other individuals or cell lines), and dendritic cell (DC) vaccines [28,31,37,38].

WTC vaccines use intact or lysed tumor cells, either with or without modifications, as a source of tumor antigens and other immunogenic factors to stimulate an immune response against cancer [39]. Autologous tumor cell vaccines are prepared using patient-derived tumor cells, typically irradiated and combined with an immunostimulatory adjuvant, then administered to the same individual. In this way, the entire spectrum of TAA is presented to the patient’s immune system. The disadvantage is the complex manufacture since the preparation of this type requires adequate tumor specimens. On the other hand, an advantage is the possibility of having a standardized and large-scale vaccine production, thus could be cost-effective [26].

DC vaccines use autologous patient-derived DCs that are loaded with peptide antigens or transfected with antigen genes, usually delivered with adjuvant molecules [28,38,39]. Conventional DCs, being the most potent APC, can activate anti-tumor immunity when given with other active immunotherapeutics [26]. The use of cell-derived vaccines can induce strong immune stimulation against several tumor antigens. On the other hand, the development of this vaccine is expensive and complex. It requires leukaphereses to isolate peripheral blood mononuclear cells from patients and ex vivo manipulation in dedicated cell factories, thus limiting the number of vaccinations [26]. Moreover, there is the need for patient-specific customization [36]. 

By exploiting the inherent immunogenicity of viruses and bacteria, they can be employed as vectors to incorporate cancer antigens by modifying their genome. Recombinant viral/bacterial vaccines can be classified into three groups: inactivated vaccines, which use killed viruses/bacteria that have been cultured in vitro, attenuated vaccines, which use weakened but not completely killed viruses/bacteria, and subunit vaccines, which use a portion of the virus/bacteria-like protein [40].

Viral vector vaccines can deliver tumor antigens to immune cells like APCs, which consequently trigger the activation of T cells and promote anti-tumor immune responses [36,41,42]. Viral vectors have low disease-causing potential and low intrinsic immunogenicity and are engineered to encode TAAs with or without immunomodulating molecules [26]. Specifically, the most commonly used viral vectors derive from adenoviruses, poxviruses, and alphaviruses. From a safety point of view, there are preferred replication-defective or attenuated versions [22]. Viral vaccines can have an oncolytic action when modified with genetic engineering, which enhances the potency of the virus to effectively replicate and kill the tumor cell [31,43,44,45]. Despite the potential immunogenicity of the vector and the need for specialized storage conditions, this type of vaccine is highly immunogenic, producing a long-lasting and specific immune response, both humoral and cell-mediated [26,36].

Antigen delivery, using a molecular platform, has been investigated due to its easier manufacturing. A tumor antigen peptide can be delivered directly as a protein or generated from DNA or RNA. DNA and RNA vaccines deliver genes in vivo to host antigen-presenting cells (APCs) to encode and express tumor antigens [26]. The tumor antigen needs to be presented on APCs in an MHC-restricted way to trigger an adaptive immune response. Then, the activated cytotoxic T-lymphocyte (CTL) will recognize the same epitope presented on the surface of the tumor cells via MHC I to eliminate the tumor cells [46,47]. The coadministration of adjuvant molecules has been demonstrated to increase vaccine efficacy [22].

DNA vaccines stimulate the host to produce antigens that trigger both humoral and cellular immune responses. DNA vaccines can incorporate multiple genes and, therefore, induce a long-term expression of several tumor antigens. Non-viral DNA vaccines consist of circular DNA plasmids encoding one or more tumor antigens and immunomodulatory molecules.

The advantages of DNA vaccine include an easy, low-cost, and cell-independent production, good stability and solubility, long-lasting immune response, and the potential for targeting multiple neoantigens [22,36]. Moreover, plasmid DNA vaccines can be designed to act as both an antigen and adjuvant [22,48]. On the contrary, DNA vaccines have a low transfection efficiency and the risk of integration into the host genome leading to reduced efficacy [26,36].

Several delivery methods have been developed, including viral vectors, mechanical techniques (microinjection, pressure, particle bombardment), electrical methods (electroporation), and chemical approaches (ionic and polymer-based) [49], such as nanoparticles, cationic conjugates, coated gold beads, and liposomal covering [50]. These delivery systems have been successful in overcoming the challenge of low immunogenicity associated with DNA vaccines, enabling targeted delivery to specific tissues or cells [49]. Electroporation (EP) is commonly used for DNA vaccine delivery but is associated with adverse effects like pain and possible muscle damage. As an alternative, lipid nanoparticles (LNPs), already used for mRNA and siRNA vaccines, show promise for DNA vaccines. Studies in mice have shown that HPV DNA vaccines encapsulated in LNPs resulted in better outcomes compared to EP, indicating that LNP-encapsulated DNA vaccines could significantly expand the vaccine market [51].

RNA vaccines deliver a mRNA that only requires translation to generate cancer antigens [52]. RNA only needs to be delivered to the cytoplasm, which makes the delivery easier than DNA vaccines [22]. Like DNA vaccines, RNA vaccines are easily produced in vitro [31]. RNA is single-stranded and can contain information to encode a few or many antigenic peptides. RNA vaccines are typically delivered in lipid nanoparticles containing adjuvant molecules [31]. The advantages of this type of vaccine are rapid development and easy modification, high immunogenicity, and cell-independent production. In addition, the RNA vaccine platform has the advantage that RNA does not integrate into the host cell genome. However, RNA is very susceptible to extracellular degradation and has the potential to induce inflammatory reactions [22,36].

Based on the interaction between the T cell receptor (TCR) and the peptide–MHC complex [22], peptide-based cancer vaccines have been developed. Peptide-based vaccines are composed of amino acid sequences containing the epitope that can trigger an immune response [46].

Peptide vaccines can be either short peptides (consisting of about 10 amino acids) or synthetic long peptides (SLP) (consisting of 25–35 amino acids) [31,46]. Short peptides can bind to MHC-I molecules in all nuclear cells, thus leading to a suboptimal CTL response [53]. Conversely, since SLP vaccines employ DC-mediated antigen presentation to activate both CD8+ T and CD4+ T cells, they are usually more immunogenic [22,54]. Peptide vaccines have many advantages, such as high specificity and safety, cell-independent production, low risk of autoimmunity, direct presentation on MHC (for short peptides), and proven clinical activity (for SLP) [36]. In addition, they are easy to produce, safe, and suitable for booster doses. As for stability, they are water-soluble and could be freeze-dried, but are also stable at room temperature; thus, they are easy to store and distribute, cost-effective; free of bacterial/viral contaminating substances, and devoid of oncogenic potential [26]. However, a peptide vaccine is prone to inducing self-tolerance and is rapidly degraded by serum/tissue peptidases; therefore, immune responses may be transient and/or low, and they need to be combined with immunogenic adjuvants [26,36].

## 2. Oropharyngeal Cancer

### 2.1. Cancer Vaccines for Viral-Induced Oropharyngeal Cancer

HPV is an oncogenic double-stranded DNA virus that is involved in the genesis of OPCs [55]. Three prophylactic HPV vaccines directed against L1 proteins of the viral capsid have been produced, namely bivalent (HPV16/18), quadrivalent (HPV6/11/16/18), and nonavalent (HPV6/11/16/18/31/33/45/52/58). However, they are ineffective in the treatment of HPV-induced OPC [56]. Indeed, prophylactic vaccines elicit the production of neutralizing antibodies that bind to the viral capsid L1 proteins and block virus entrance into host cells; however, these proteins are not expressed in already infected basal epithelial cells, thus preventing the identification or eradication of already infected cells [57].

Nevertheless, HPV-positive cancer cells generate non-self antigens, which could become potential targets for a vaccination strategy [58]. Ideal HPV-derived antigens are the oncoproteins E6 and E7 because they are expressed constitutively, at high levels, and exclusively in cancer cells. In addition, these oncoproteins are also essential for the onset and maintenance of malignancy [26].

**Figure 2:**
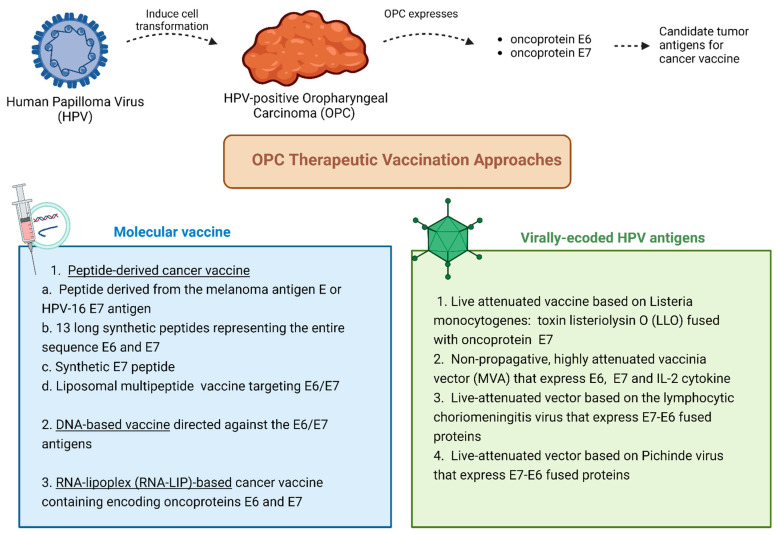
Different therapeutic approaches for HPV-positive oropharyngeal cancer.

Several clinical trials are investigating the use of different tumor antigens and delivery platforms in the treatment of HPV-induced HNC, including OPCs [46] (Figure 2).

Among peptide-based cancer vaccines, we can mention the “trojan” peptide vaccine based on a peptide derived from the HPV-16 E7 antigen [59,60], ISA101 vaccine that includes 13 long synthetic peptides of 25 to 35 amino acids encompassing the entire sequence of HPV-16 E6 and E7 [61], DPX-E7, which is a synthetic peptide consisting of amino acids 11 through 19 of the viral oncoprotein HPV-16 E7 [57,62], and a liposomal multipeptide therapeutic vaccine targeting HPV-16 E6/E7 (PDS0101) [57].

For OPCs, a DNA-based vaccine encoding HPV18 E6/E7 antigens plus IL12 (INO-3112) [63] and an RNA-lipoplex (RNA-LIP)-based cancer vaccine containing a mRNA encoding oncoproteins E6 and E7(BNT113) [62] are currently under investigation.

Concerning vaccines derived from infective agents, a live attenuated vaccine based on Listeria monocytogenes, ADXS11, has been produced with a fragment of the toxin listeriolysin O (LLO) that is fused to the oncoprotein HPV-16 E7 [64,65]. In addition, we can mention TG4001 vaccine, which is a non-propagative, highly attenuated vaccinia vector (MVA) that was engineered to express the coding sequences of E6 and E7 and IL-2 cytokine [66], and HB-201 and HB-202, which are two live-attenuated vectors based on the lymphocytic choriomeningitis virus and Pichinde virus, respectively, that express E7-E6 fused proteins and infect APCs to induce tumor-specific T cell responses [26].

### 2.2. Vaccine-Based Immunotherapy Clinical Trials for HPV-Induced Oropharyngeal Cancer

The development of therapeutic vaccines targeting HPV-related antigens in HNSCC is a rapidly evolving field with multiple clinical trials investigating their efficacy and safety (Table 2).

The HPV-16 E6/E7 therapeutic vaccination, ISA101 (ISA101b), administered subcutaneously, has been tested in combination with various agents. In a phase II clinical trial with CD137 (4-1BB, TNF) agonist utomilumab, the study was closed due to slow patient accrual (NCT03258008). Another phase II trial is currently recruiting to evaluate the combination of ISA101b with pembrolizumab, cisplatin, and RT for patients with a locally advanced, intermediate-risk, HPV-associated HNSCC (NCT04369937). In a phase II trial, of the 24 enrolled patients (22 with HPV-positive OPC), ISA101b combined with nivolumab showed an overall response rate (ORR) of 33%, with a median progression-free survival (PFS) of 2.7 months and a median OS of 15.3 months (NCT02426892). Grades (G) 3/4 Adverse Events (AEs) occurred in two patients (asymptomatic grade 3 transaminase level elevation in one patient and grade 4 lipase elevation in 1 patient), requiring discontinuation of nivolumab therapy.

Patients with confirmed HPV16+ R/M OPC who were naïve to anti-PD-1 therapy in both first- and second-line settings were randomized to receive either ISA101b (100 µg/peptide subcutaneously on days 1, 29, and 50) or a placebo, in combination with cemiplimab (350 mg intravenously every 21 days) for up to 24 months or until disease progression or treatment discontinuation. In the ISA101b arm, the ORR was 25.3%, compared to 22.9% in the control arm (not significant). Severe AEs (SAEs) were reported in 33% of patients receiving ISA101b versus 31.6% in the control group. Patients with a CPS ≥ 20 who received cemiplimab in combination with three doses of ISA101b demonstrated a significantly improved ORR and OS compared to those in the control arm. In contrast, patients with CPS < 20 did not benefit (NCT03669718).

The ADXS11-001 vaccine, based on live attenuated Listeria monocytogenes targeting HPV16 E7, administered intravenously, demonstrated significant immune responses in a window of opportunity trial in 15 surgically elected HPV-positive OPC patients before transoral surgery, showing a notable increase in HPV-specific T cell response (NCT02002182). The second primary outcome measure is the G3/G4 toxicity assessed according to NCI Common Terminology Criteria for Adverse Events (CTCAE) 4.0 criteria, assessed up to 30 days after surgery, reporting a 55.6% in the vaccination arm vs. 16.7% in the control arm.

The PRGN-2009 vaccine, utilizing a novel gorilla adenovirus vector targeting HPV16 and 18 E6/E7, administered subcutaneously, was tested alone and in combination with bintrafusp alfa, a bifunctional TGF-β “trap”/anti-PD-L1 fusion protein. In a phase I trial, in the dose escalation part, 6 patients received PRGN-2009 whereas in the combination part, 11 high-risk HPV-positive patients received PRGN-2009 with bintrafusp alfa achieving an ORR of 30% across all cohorts. The median OS was 7.4 months for the dose escalation phase and 12.5 months for the combination. No G3/4 AEs were reported in the dose escalation while 27% were revealed in the combination treatment, across all cohorts (NCT04432597).

The DPX-E7 synthetic peptide-based vaccine was evaluated alone or with cyclophosphamide, in HPV-positive OPC, cervical, and anal cancers, positive for HLA-A*02, showed no ORR with only stable disease as the best response with varying adverse events into the different phases of the study. No DLT was observed. The proportion of responders’ patients who achieved an increase in the number of CD8+ T cells in the peripheral blood and tumor tissue was 16.7% (NCT02865135).

The RNA-lipoplex (RNA-LIP)-based mRNA vaccine BNT113, encoding HPV-16 E6 and E7 (administered intravenously) combined with pembrolizumab, is evaluated in a recruiting randomized phase II trial enrolling patients with HPV 16-positive and PD-L1 expressing R/M HNSCC as first-line therapy. Initial results indicate manageable safety profiles. The G3/4 AEs (pyrexia, hypercalcemia, pleural effusion, shaking/rigors) were observed in 3 out of 12 patients (25%) who completed the safety run-in phase, with pyrexia and shaking/rigors considered treatment-related (NCT04534205).

A peptide-based vaccine, PDS0101, which is a liposomal HPV-16 E6/E7multipeptide vaccine that upregulates type 1 IFN and induces T-cell activation (given subcutaneously), is being evaluated in a phase II trial, combined with pembrolizumab. VERSATILE-002 (NCT04260126) is an open-label, non-randomized study evaluating the combination of PDS0101 and pembrolizumab in subjects with HPV16-positive R/M HNSCC in two cohorts: ICI-naïve expressing PD-L1 ≥ 1 and ICI-refractory. In the ICI-naïve cohort, the ORR was 26.5%, the median PFS was 10.4, and the estimated 12-month OS of 87.1%. The G3/4 AEs were 13% and 4% in pts ICIs naïve and ICIs pre-treated, respectively (NCT04260126).

The triple combination of PDS0101, M9241, an immune-cytokine targeting DNA release from necrotic tumor cells, and bintrafusp alfa showed in 30 patients with advanced HPV16-positive cancers (9 cervical, 2 vaginal/vulvar, 6 anal, 13 OPC) promising activity with an ORR of 6/22 (27%) in ICIs-treated and 7/8 (88%) in ICIs-naive advanced HPV16-positive cancers (NCT04287868). Across all cohorts, the G3/4 AEs were reported in 43%. 6/8 (75%) pts with checkpoint naïve disease and 17/22 (77%) pts with checkpoint refractory disease are alive after a median of 17 and 12 months follow up respectively.

The DNA-based MEDI0457 vaccine, targeting HPV16/18 E6/E7, with an intramuscular administration, and combined with durvalumab, in a phase I/IIa trial, demonstrated in R/M HNSCC an ORR of 22.2% with three complete responses, three partial responses and G3/4 AEs in 17% (AST and ALT increased and myocarditis causing discontinuation). Peripheral HPV-specific T cells and tumoral CD8+ T cells were increased (NCT03162224).

Additionally, a phase IB clinical trial of intratumoral talimogene laherparepvec (TVEC, HSV-1) (intradermal injection) combined with pembrolizumab showed an ORR of 16.7% in R/M HNSCC, refractory to platinum-based chemotherapy, with a median duration of response (DOR) of 45.9 months. In total, 14% of patients experienced G3/4 AEs. One DLT of T-VEC-related fatal arterial hemorrhage was reported. The median PFS and OS were 3 and 5.8 months, respectively (NCT02626000).

Lastly, the CUE-101 vaccine, featuring a peptide epitope from HPV16 E7 and IL-2 molecules (intravenous administration), was evaluated in a phase I trial, alone or combined with pembrolizumab in HPV 16 + R/M HNSCC. An ORR of 47% and a mPFS of 5.8 months were observed for patients treated with combination therapy as first-line therapy. A median OS of 20.8 was observed in patients treated with CUE-101 monotherapy as post-platinum/ICIs therapy. The most frequent G3 AEs include lymphocyte count decreased (7.6%), anemia (6.3%), decreased appetite (5.1%), and infusion-related reactions (5.1%) (NCT03978689).

## 3. Nasopharyngeal Cancer

### 3.1. Cancer Vaccines for Viral-Induced Nasopharyngeal Cancer: An Overview

NPC is associated with EBV infection in over 95% of cases. EBV has been associated also with the development of various lymphoid and epithelial cancers, including Burkitt lymphoma (BL), Hodgkin lymphoma (HL), post-transplant lymphoproliferative disorders (PTLD), and gastric carcinoma (GC) [77].

EBV-positive NPCs express two interesting proteins: the Epstein–Barr nuclear antigen 1 (EBNA1), which is involved in the maintenance of the viral DNA in episomal form and has several epitopes stimulating a CD4+ T-cell response, and the latent membrane protein 2 (LMP2), which is has a transmembrane location in infected cells and is involved in cell growth activity in epithelia and has many epitopes stimulating a CD8+ T-cell response [46,78,79].

**Figure 3:**
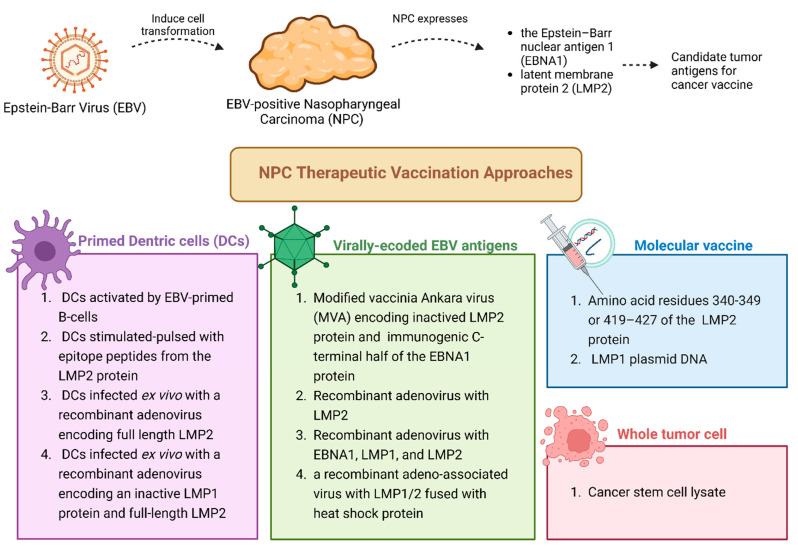
Different therapeutic approaches for EBV-positive nasopharyngeal cancer.

Therapeutic vaccines for NPC have been developed using cell-derived, virus, and peptide platforms [46] (Figure 3).

The first group of vaccines is based on autologous DCs that can be activated by EBV-primed B-cells [80], stimulated-pulsed with epitope peptides from the LMP2 protein [81,82], or infected ex vivo with a recombinant adenovirus encoding full-length LMP2 [83] or an inactive LMP1 protein and full-length LMP2 [84].

A second class of NPC vaccines is based on virally encoded EBV-antigens. A vaccine based on the modified vaccinia Ankara virus (MVA) that encodes an inactive form of the LMP2 protein and the immunogenic C-terminal half of the EBNA1 protein has been developed [79]. In addition, vaccines based on recombinant adenovirus with LMP2 [85], with EBNA1, LMP1, and LMP2 (AdE1-LMPpoly vector) [86] (Ad-SAVINE) [87] and a vaccine based on a recombinant adeno-associated virus with LMP1/2 fused with heat shock protein (rAAV-LMP2/1-hsp) [88] have been produced [89].

Other NPC therapeutic vaccination approaches include cancer stem cell lysate [89], peptide cancer vaccines containing amino acid residues 340–349 or 419–427 of the EBV LMP2 protein (LMP-2:340–349, LMP-2:419–427) [89], and LMP1 plasmid DNA [90].

### 3.2. Vaccine-Based Immunotherapy Clinical Trials for EBV-Induced Nasopharyngeal Cancer

Several clinical trials of virus vector vaccines targeting EBV-associated malignancies have been completed (Table 3). Most vaccines against EBV-induced NPC have utilized the viral antigens EBNA1 and LMP2 as targets, following the intradermal administration route.

One study evaluated the MVA-EL vaccine targeting both EBNA1/LMP2 antigens in 16 patients with EBV-induced NPC who were in complete remission after the first-line treatment. No G3/G4 AEs were reported and the highest dose (5 × 10(8) pfu) was recommended for investigation in current phase IB and II trials (NCT01147991).

A similar phase I trial evaluated the same vaccine in 18 Chinese patients after standard treatment. Fifteen of the eighteen patients experienced an increase in antigen-specific T cells in an ELISpot test after vaccination, and 5.5% reported G3/G4 AEs (NCT01256853).

Further research into the MVA-EBNA1/LMP2 vaccine included a phase Ib trial with 22 patients in remission or with current disease, where the primary outcomes were safety and IR (NCT01800071). A phase II trial with 25 patients who had persistent, recurrent, or metastatic NPC is evaluating the efficacy of the same vaccine, with the clinical benefit rate as the primary endpoint (NCT01094405).

A Chinese clinical trial involved the use of autologous DC vaccines primed to induce LMP2-positive cell recognition [82]: 16 HLA-A2 patients with stage II-III NPC received vaccinations with autologous DCs pulsed with a HLA-A2-restricted LMP2A peptide. The vaccination was combined with (C)RT. A significant increase in serum interferon-gamma and interleukin-2 levels was observed post-vaccination, along with a rise in the percentage of CD4+ T-cells and natural killer cells. Notably, serum EBV DNA levels significantly decreased in the nine patients who demonstrated a skin response to the LMP2A peptide in a delayed hypersensitivity test. The vaccine was well-tolerated, with no recurrence detected during the follow-up.

A recruiting phase I/II study examined the safety and efficacy of LMP2 peptide vaccines in 99 patients with locally controlled anaplastic NPC (NCT00078494). A phase I trial is currently investigating a mRNA vaccine in nine patients with advanced EBV-positive tumors after second-line therapy (NCT05714748).

A separate phase II trial examined these transduced DCs without celecoxib in 16 patients, reporting an ORR of 19% and no G3/G4 AEs [84]. Lastly, a phase I trial evaluated rAd5-EBV-LMP2-transduced autologous DCs in 24 patients with regional advanced disease: the vaccine was generally well-tolerated and the high dose (2 × 10^11^ vp) is recommended to be adopted in phase II studies.

Advancements in vaccine development, including viral vector vaccines, adjuvants that stimulate CD4+ and CD8+ T-cell responses, and structural biology techniques to identify more immunogenic conformations of viral proteins, as well as mRNA vaccines and novel methods for mucosal vaccines, offer hope for further improvement of therapeutic vaccines targeting the EBV.

## 4. Discussion: Clinical Challenges and Ethical Considerations

Therapeutic vaccines are considered a promising yet still developing approach for HNC, including both HPV-related and EBV-related cases. While these vaccines can elicit an immune response against tumor antigens, their clinical efficacy remains limited when used as monotherapy. However, early findings indicate that combining therapeutic vaccines with ICIs, such as anti-PD-1 therapies, may enhance their effectiveness. Despite their potential, several challenges persist. A significant proportion of HNC cases exhibit an immune-depleted state, which reduces the effectiveness of many vaccination strategies. Additionally, operational barriers, such as vaccine manufacturing, storage, distribution logistics, and cost, can impede widespread implementation. The transition from clinical trials to routine clinical practice remains a pivotal yet underexplored challenge in vaccine-based immunotherapy for HPV- and EBV-associated cancers [36]. Clinical trials often involve highly selected patient cohorts, with strict inclusion criteria, which may not represent the heterogeneity of real-world populations. In routine practice, factors such as patient comorbidities, treatment compliance, and socioeconomic disparities can introduce variability in outcomes. Real-world data from post-marketing surveillance should be emphasized to optimize vaccine deployment strategies, inform clinical guidelines, and address gaps in accessibility.

Ethical considerations play a fundamental role in both the development of vaccines and the clinical application of vaccine-based immunotherapy [94]. The ethical framework guiding the development of therapeutic cancer vaccines in clinical trials must adhere to fundamental bioethical principles.

Ensuring fair access to these vaccines globally is crucial, preventing disparities between wealthy and resource-limited countries. Additionally, ensuring that enrolled patients fully understand the potential risks and benefits is paramount, particularly in early-phase trials where long-term outcomes remain uncertain [95]. Patient selection criteria should be inclusive to ensure diverse representation and avoid biases that could limit the generalizability of findings [96].

Therapeutic vaccines for HPV and EBV-related HNC present significant differences both in terms of development and mechanisms of action [97]. Anti-HPV vaccines are designed to treat HNC caused by persistent HPV infections, particularly types 16 and 18, which are responsible for many OPCs. Currently, therapeutic HPV vaccines are in clinical trials and show promising results, but long-term data are still limited. Nucleic acid and mRNA-based vaccines are gaining attention as potentially more effective in enhancing immunity against tumors. EBV is linked to NPC, which is, compared to HPV-related cancers, less widely recognized globally despite its high incidence in certain regions [98]. Although therapeutic EBV vaccines are still under development, the approach focuses on stimulating the immune system to recognize and destroy EBV-infected cells. In summary, while therapeutic HPV vaccines for HNC have shown faster and more concrete progress, those targeting EBV are still in the exploratory phase. Both types of vaccines represent potential promise, but each faces unique challenges in stimulating an effective immune response against tumors associated with their respective viruses. Optimizing antigen delivery platforms, enhancing adjuvant formulations, and overcoming immune tolerance are crucial for maximizing the clinical potential of these vaccines.

In conclusion, vaccine-based therapies for HPV-related OPC and EBV-related NPC have shown good tolerability and promising results, particularly when combined with other immunotherapeutic approaches (e.g., ICIs), in the early-phase clinical trials. These preliminary findings suggest a potential clinical benefit in treating these cancers. However, larger clinical trials and better patient selection strategies are required to fully determine the future role of therapeutic vaccines in the treatment of HNC. Such studies are essential to determine long-term effectiveness and optimize vaccine strategies and potential combinations. Addressing both clinical and ethical challenges are necessary to validate their potential to improve virus-related cancer care.

## Figures and Tables

**Table 1:** Classification of tumor antigens.

Cancer Antigen Source	Categories	Target Types	Description	Tumor Specificity	Central Tolerance	Prevalence in Multiple Patients	Examples
Tumor cell	Tumor-associated antigens (TAAs)	Differentiation antigens	Antigens expressed during tissue differentiation	Variable	High	High	Melan A, CD19
		Overexpressed antigens	Antigens overexpressed on tumor cells compared to normal cells	Variable	High	High	HER2, TROP2
		Cancer testis antigens	Antigens limitedly expressed on testes, fetal ovaries, and trophoblast	Good	Low	High	MAGE-A3, NY-ESO-1
	Tumor-specific antigens	Private-personalized neoantigens	Antigens resulting from somatic mutation of patient-specific mutated genes	Ideal	None	Low	Numerous
		Shared-public antigens	Antigens resulting from somatic mutation of recurrently mutated genes	Ideal	None	High	KRAS, p53
Virus	Oncoviral antigens		Antigens expressed on cancer cells infected with an oncovirus	Ideal	None	High	EBV LMP, HPV E6/7

Table modified from [31].

**Table 2:** Clinical trials evaluating vaccines-based treatment in HPV-positive OPCs.

	Class	Target Antigen	Phase	Population/ Setting	Administration	N	ORR	G3/G4 AEs	Other Results	Reference
ISA101b + CD137 (4-1BB, TNF) agonist utomilumab	Peptide	HPV16 E6/E7	II	HPV-positive OPCs	sc	3	NA	NA	trial closed for infeasibility due to slow accrual	NCT03258008
ISA101b + pembrolizumab + cisplatin+ radiotherapy	Peptide	HPV16 E6/E7	II	Intermediate risk HPV-positive HNSCC	sc	50	NA	NA	NA	NCT04369937
ISA101b + nivolumab	Peptide	HPV16 E6/E7	II	Incurable HPV16+ cancers	sc	24 (22 OPC)	33%	46%	mPFS: 2.7 mo; mOS 15.3 mo	NCT02426892 [61,67]
ISA101b + cemiplimab (vs. cemiplimab)	Peptide	HPV16 E6/E7	II	First and second line anti-PD-1 naïve HPV16+ OPC patients	sc	91 (in the combination arm)	25%	33%	In CPS ≥ 20: mOS not reached (28.1, -). In CPS < 20: shorter OS	NCT03669718 [68]
ADXS11-001	Live attenuated Listeria Monocytogenes	HPV16 E7	II	Surgically elected HPV+ OPC (Prior to Robot-Assisted Resection)	IV	15	NA	56%	0.625 number of pts with a >2-fold increase in HPV-specific T-cell response	NCT02002182
PRGN-2009 alone or in combination with bintrafusp alfa	novel gorilla adenovirus	HPV 16 and 18 E6/E7	I	HPV-positive cancers	sc	17	30% (all cohorts)	0% (dose escalation, all cohorts) 27% (combination cohort, all cohorts)	mOS: 7.4 mo for dose escalation; mOS: 12.5 mo for combination.	NCT04432597 [69]
DPX-E7 alone or with cyclophosphamide	synthetic peptide-based vaccine	HPV16 E711-19	I/II	HPV-positive OPC, cervical and anal cancers (positive for HLA-A*02)	n.d.	11	0%	33% (phase Ib) 0% (phase II cohort 1) 50% (phase II cohort 2)	17% (0.6 to 64.1) changes in responders DLT: 0%	NCT02865135
BNT113 plus pembrolizumab (Vs pembrolizumab alone)	RNA	HPV16 E6/E7	II	R/M HPV16-positive and PD-L1 expressing HNSCC	IV	12	NA	25%	NA	NCT04534205 [70]
PDS0101 plus pembrolizumab	Peptide	HPV16 E6/E7	II	R/M HPV-positive HNSCC (ICI naive PD-L1 ≥ 1 or ICI-pre treated)	sc	48	26%. (ICI naïve)	13% (ICI naïve) 4% (ICI pre-treated)	mPFS: 10.4mo, 1-yr OS 87% (ICI naïve)	NCT04260126 (VERSATILE-002) [71]
PDS0101+ tumor-targeting interleukin-12 (IL-12) fusion protein M9241 (NHS-IL12) + bintrafusp alfa (Triplet)	Peptide	HPV16 E6/E7	II	Advanced HPV16-positive cancers	sc	30 (13 OPC)	7/8 (88%) (checkpoint naïve ); 6/22 (27%) (checkpoint refractory)	43% (all cohorts)	6/8 (75%) pts with checkpoint naïve disease and 17/22 (77%) pts with checkpoint refractory disease are alive after a median of 17 and 12 months follow up respectively.	NCT04287868 [72]
MEDI0457 + durvalumab	DNA	HPV16 E6/E7 HPV18 E6/E7	I/IIa	R/M HNSCC	IM	35	22%	17%	Tumor-infiltrating CD8+ T cells and peripheral HPV-specific T cells were increased	NCT03162224 [73]
Intratumoral talimogene laherparepvec (TVEC, HSV-1) + pembrolizumab	oncolytic	genetically modified herpes simplex virus-1	IB	R/M HNSCC refractory to platinum-based chemotherapy	intradermal	36	17%	14%	DCR 38.9%; mDOR: 45.9 mo; mPFS:3.0 mo; mOS:5.8 mo.	NCT02626000 [74]
CUE-101 (‘T cell engagers’: HLA-A*0201 complex + E7 epitope + IL-2 molecules) +/− Pembrolizumab	peptide epitope derived from the HPV16 E7 protein and 4 molecules of attenuated human interleukin-2 (IL-2)	HPV16 E7-pHLA-IL2-Fc fusion protein	I	HPV16+ R/M HNSCC	IV	80 (49 in monotherapy and 31 CUE-101 plus pembrolizumab)	Combo 47% Mono 5.3%	8% lymphocyte count decreased; 6% anemia; 5% decreased appetite; 5% infusion-related reactions	Combo: mPFS of 5.8 mo Mono: mOS of 20.8 mo	NCT03978689 [75,76]

Abbreviations: N: number of enrolled patients; ORR: Overall Response Rate; G3/G4 AEs: grade 3/4 Adverse Events; sc: subcutaneous; m: median; pts: patients; PFS: progression-free survival; OS: overall survival; OPC: oropharyngeal cancer; DCR: disease control rate; NA: not assessed; n.d.: not disclosed; R/M: recurrent/metastatic; HNSCC: head and neck squamous cell carcinoma; IV: intravenous; IM: intramuscular; RP2D: recommended phase II dose; DLT: dose-limiting toxicity; ICIs: immune checkpoint inhibitors; mo: months; +: plus; *: Standard separator in HLA nomenclature used to distinguish specific alleles.

**Table 3:** Completed clinical trials evaluating vaccines-based treatment in EBV-positive NPCs.

Vaccine + Combined Therapy	Class	Target Antigen	Phase of Study	Population/ Setting	N	Administration	G3/G4 AEs	Other Results	Reference
MVA-EL	Live (MVA virus)	EBNA 1/LMP2	I	EBV-induced NPC in CR after first-line treatment	16	intradermal	0%	to assess changes in EBV genome levels and EBNA1-specific antibodies in plasma	NCT01147991 [79]
MVA-EL	Live (MVA virus)	EBNA 1/LMP2	I	EBV-induced NPC in CR or unconfirmed CR	18	intradermal	5%	to determine the dose for subsequent efficacy trials	NCT01256853 [91]
MVA-EBNA1/LMP2	Live (MVA virus)	EBNA 1/LMP2	Ib	EBV-induced NPC in remission or with current disease for whom no standard therapy is required	22	intradermal	NA	NA	NCT01800071
MVA-EBNA1/LMP2 vaccine	Live (MVA virus)	EBNA 1/LMP2	II	R/M NPC with residual EBV DNA following conventional therapy	25	NA	NA	clinical benefit rate	NCT01094405
DC vaccine	Autologous DC	LMP2	NA	stage II-III NPC	16	NA	0%	lymphocyte subsets, serum cytokines, EBV-DNA levels, the delayed-type hypersensitivity (DTH) responses	[82]
LMP-2:340–349 or LMP-2:419–427	peptide	LMP2	I/II	locally controlled anaplastic NPC	99	intradermal	NA	NA	NCT00078494
mRNA Vaccine	mRNA vaccine	none	I	EBV-positive advanced malignant tumors after failure of second-line standard therapy	9	IM	0%	AEs; PFS; OS	NCT05714748 [92]
Ad5F35-LMP1/LMP2-transduced autologous DCs	Autologous DCs transduced with adenovirus	LMP1/LMP2	II	R/M NPC	16	intradermal	0%	-	[84]
rAd5-EBV-LMP2	Autologous DCs transduced with adenovirus	EBV-antigen	I	regional advanced NPC	24	IM	0%	-	[93]

Abbreviations: N: number of enrolled patients; G3/G4 AEs: grade 3/4 Adverse Events; PFS: progression-free survival; OS: overall survival; NPC: nasopharyngeal cancer; CR: complete response; NA: not assessed; n.d.: not disclosed; R/M: recurrent/metastatic; HNSCC: head and neck squamous cell carcinoma; IM: intramuscular.

## Data Availability

The data used to support the findings of this study are available upon request to the corresponding author.
